# Supplementary material for: Predicting oxygen levels in microbial habitats using a metagenome-based approach
Source: mSystems. 2026 Jun 16;11(7):e00545-26. doi: 10.1128/msystems.00545-26 (PMC13386939; doi:10.1128/msystems.00545-26)
Supplement: Supplemental Figures — Figures S1 to S8. [file msystems.00545-26-s0001.docx]

**Supplemental figures for:**

**Predicting oxygen levels in microbial habitats using a metagenome-based approach**

Clifton P. Bueno de Mesquita^1,2#^, Elías Stallard-Olivera^1,2^, Noah Fierer^1,2#^

^1^ Cooperative Institute for Research in Environmental Sciences, University of Colorado, Boulder, Colorado, USA

^2^ Department of Ecology and Evolutionary Biology, University of Colorado, Boulder, Colorado, USA

^#^ Corresponding authors: [cliff.buenodemesquita@colorado.edu](mailto:cliff.buenodemesquita@colorado.edu), [noah.fierer@colorado.edu](mailto:noah.fierer@colorado.edu)

Keywords: oxygen, oxygen tolerance, aerobes, anaerobes, facultative, bioindicators

Running title: Predicting oxygen levels in microbial habitats


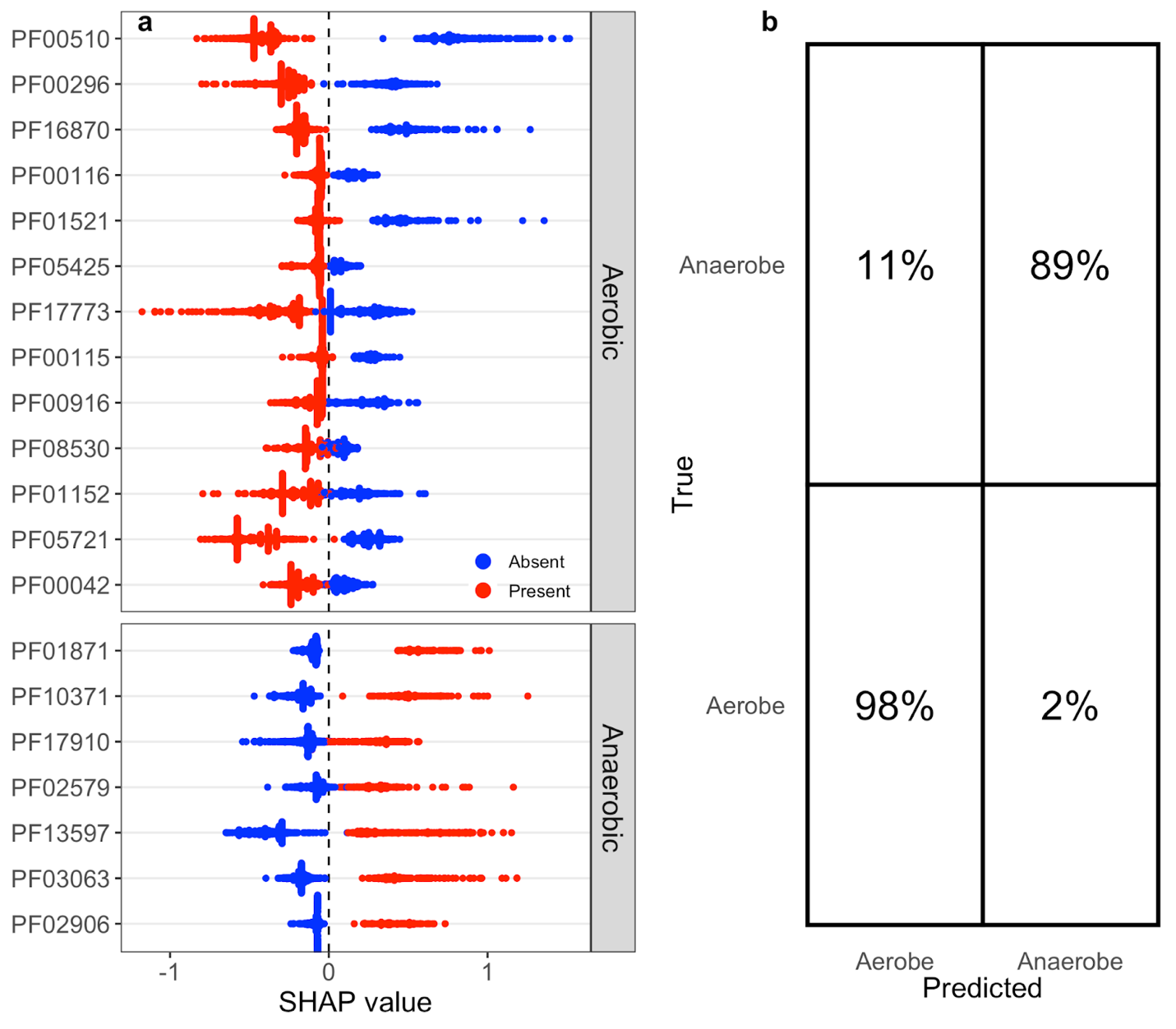


Figure S1. a) Average SHAP values of the 20 selected Pfams (13 aerobic indicators, 7 anaerobic indicators) from ensemble machine learning classification modeling. For Pfam names and other information, see Table 1. b) Confusion matrix of the genome-level ensemble machine learning model predicting whether a genome comes from an aerobe or anaerobe based on BacDive data (training n = 4416, testing n = 1104).

Figure S2. Relationship between the percent of aerobe genomes in simulated metagenomes, and the ratio of the RPK of aerobic indicator genes to anaerobic indicator genes, across two ranges of sequencing depths. The model used for prediction in OxyMetaG is the one trained with data from 26 to 41 million reads.

Figure S3. Comparison of predicted oxygen levels across the 16-habitat dataset (Figure 3) using two different DIAMOND BLASTX hit cutoffs. n = 173 of the 203 samples from Figure 3 that had predictions using both cutoffs (with stringent cutoffs, there were 20 samples that did not have at least one aerobic indicator Pfam and at least one anaerobic indicator Pfam). The dashed black line represents the 1:1 line and the solid blue line is the linear regression line. The two predictions were strongly and significantly positively correlated (R^2^ = 0.99, p < 0.001). Default cutoffs: percent identity ≥ 60, e-value < 0.001, and bitscore ≥ 50. Stringent cutoffs: percent identity ≥ 60, e-value < 1e-10, bitscore ≥ 60, and amino acid alignment length ≥ 40. 100% and 0% oxygen levels indicate completely oxic and completely anoxic environments, respectively.

Figure S4. Predicted oxygen levels across climate classes in a) Australia and b) the United States. The x-axis within each panel is sorted from left to right by mean predicted percent aerobes. Insets show histograms; note the difference in axes scales between panels. Climate class did not significantly affect the predicted relative abundance of aerobic bacteria (ZIBR, p > 0.05). 100% and 0% oxygen levels indicate completely oxic and completely anoxic environments, respectively.

Figure S5. Predicted oxygen levels were significantly negatively associated with gravimetric water content in both datasets according to zero-inflated beta-regression (p < 0.05). Note that of the 331 Australian soils analyzed in this study, only 247 included data on gravimetric water content and are included in this figure. 100% and 0% oxygen levels indicate completely oxic and completely anoxic environments, respectively.


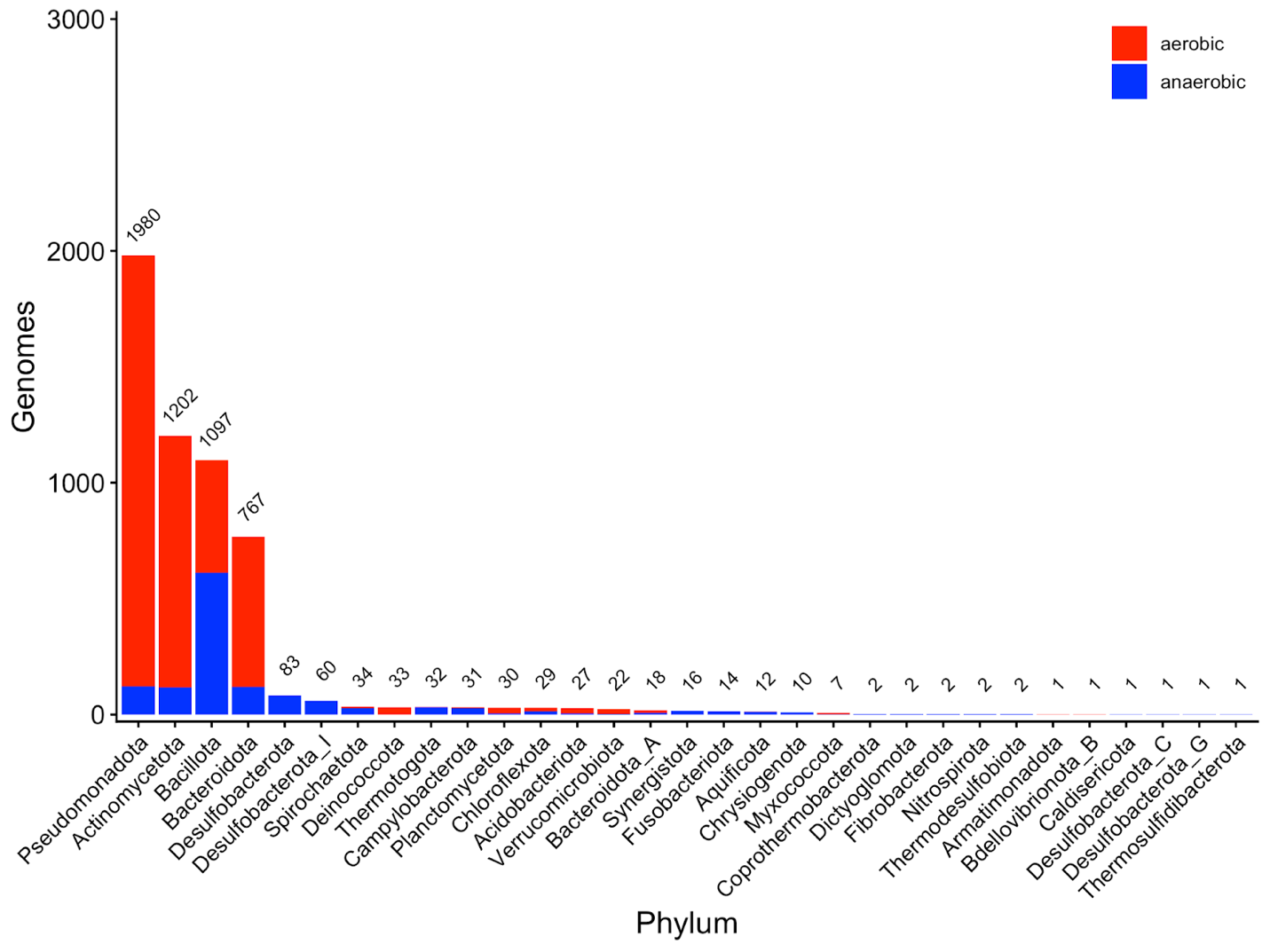


Figure S6. Number of genomes of each phylum (according to GTDB r226 taxonomy) and each oxygen tolerance category (BacDive), n = 5520. These genomes were inputs in an ensemble machine learning model to predict oxygen tolerance from Pfam presence. Numbers above the bars represent the number of genomes in each phylum.

Figure S7. Number of genomes of each phylum (according to GTDB r226 taxonomy) classified as “facultative anaerobe” or “facultative aerobe” in BacDive in April 2026, n = 1655. Random subsets of these genomes, along with aerobe and anaerobe genomes (Figure S6) were used in the metagenome simulations that are presented in Figure 2. Numbers above the bars represent the number of genomes in each phylum.

Figure S8. Assessment of the effect of sequencing depth on predicted oxygen levels. No prediction is made if 0 aerobic Pfams or 0 anaerobic Pfams are detected, which was the case until 4k reads for the human gut sample, 8k reads for the deep lake sample, and 256k reads for the surface lake sample. Note that the human gut sample did not have 32.8 million bacterial reads. 100% and 0% oxygen levels indicate completely oxic and completely anoxic environments, respectively.
